# Entropy generation analysis for MHD flow of water past an accelerated plate

**DOI:** 10.1038/s41598-021-89744-w

**Published:** 2021-06-07

**Authors:** Tarek N. Abdelhameed

**Affiliations:** 1 Basic Engineering Sciences Department, College of Engineering, Majmaah University, Majmaah, 11952 Saudi Arabia; 2 Mathematics Department, Faculty of Science, Beni-Suef University, Beni-Suef, 62514 Egypt

**Keywords:** Engineering, Nanoscience and technology

## Abstract

This article examines the entropy generation in the magnetohydrodynamics (MHD) flow of Newtonian fluid (water) under the effect of applied magnetic in the absence of an induced magnetic field. More precisely, the flow of water is considered past an accelerated plate such that the fluid is receiving constant heating from the initial plate. The fluid disturbance away from the plate is negligible, therefore, the domain of flow is considered as semi-infinite. The flow and heat transfer problem is considered in terms of differential equations with physical conditions and then the corresponding equations for entropy generation and Bejan number are developed. The problem is solved for exact solutions using the Laplace transform and finite difference methods. Results are displayed in graphs and tables and discussed for embedded flow parameters. Results showed that the magnetic field has a strong influence on water flow, entropy generation, and Bejan number.

## Introduction

Entropy plays a very important role in fluid dynamics. The second law of thermodynamics totally revolves around the entropy concepts and entropy generation. The feasibility of a process and its efficiency are directly related to entropy generation in the process. Since entropy generation is much important and happens in all flow processes because of friction in fluids, therefore, in this paper, we study entropy generation in (MHD) the flow of water which is taken as a counter-example of a Newtonian fluid, flows over a second-order accelerated plate taken in the vertical direction. One of the useful applications of entropy generation in real life/world is in the design of systems that rely on heat transfer. Entropy tells us in a way, the best we can do to avoid thermal energy losses.

Amongst the various researchers working on entropy generation, the most famous is true, Adrian Bejan, who has published many kinds of research on entropy generation and the second law of thermodynamics with applications in various fields, see for example^1–7^.

The importance of entropy generation-EG (second law of thermodynamics-SLTD) in examining heated fluid flows in several engineering devices of practical use and heat related systems has become significant. For example, in thermal analyses, one can see that a worth paying attention part of energy is wasted during the heat transfer and not producing the desired result. Indeed, the scientists were not happy with such a great loss of energy and finally they realized that such energy losses can be removed or minimized by properly designing the heat system. Entropy generation-EG can be produced by several means for instance, heat transfer in thermal systems. Some important key sources amongst the many others, from which Entropy generation-EG in thermal systems include viscous dissipation, chemical reaction, mass transfer, heat transfer, and electrical conduction^8^. In the interesting work of Awed^9^, he introduced a new definition of the Bejan number(BN). The BN is indeed, useful in several cases, as it can clearly provide evidence related the dominance of a strong magnetic field and corresponding fluid friction entropy via heat transfer, or vice versa. Saouli and Aïboud-Saouli^10^ examined entropy generation-EG in a liquid film such that it is falling along with plate inclided along the plane. A great work for the interesting analysis of entropy generation-EG for the famous Tiwari and Das model was developed by Sheremet et al.^11^. In their study the carefully examined the process and examined some interesting and important computational results for finding the solution to their problem. In their investigation, they examined carefully that addition of nanoparticles in a regular base fluid, the heat transfer rate enormously increased and, then consequently, the cavity of convective flow was found much smaller. For turbulence-forced convection, entropy generation-EG was discussed in details in an excellent and interesting work of Ji et al.^12^. However, recently, Qing et al.^13^ examined entropy generation-EG for non-Newtonian fluid of Casson model conating nanoparticles inside a regular base fluid in the presence of a strong magnetic field. The analysis was done over a porous surface with a stretching or shrinking sheet to examine MHD flow. A strong comupational technique known as successive linearization method (SLM) was used for solving a strong system of equations and highlighted the influence of various parameters on velocity and temperature. Sheikholeslami et al.^14^, numerically examined the impact of Lorentz forces on Fe_3_O_4_-water ferrofluid entropy and exergy treatment within a permeable semi annulus by applying a strong numerical scheme.

In all the above problems, entropy generation analysis was done using the numerical treatment. Indeed the entropy generation problem via exact treatment is very rare due to the complex mathematical calculi. However, limited studies are available in this direction, including the work of Saqib et al.^15^, in which they investigated entropy generation for generalized nanofluids via exact solution treatment. In this paper, the main objective was to examine entropy for fractional partial differential equations. They first formulated the problem and then solved for exact solutions fluid problem with plots and physical interpretations. They noted that the classical solutions can be obtained in a limiting sense for the unit value of the fractional parameter. Khan et al.^16^ applied the classical approach for both the formulation and solution and examined the entropy generation in an unsteady MHD flow with a combined influence of heat and mass transfer through a porous medium where the plate they consider is isothermal and ramped wall temperature.

Numerical methods or see for example Refs.^17–30^ and any other was used in many papers^31–43^ however, very limited studies are reported in which exact and numerical solutions are simultaneously obtained. Therefore, the main task here is to obtain exact and numerical solutions for the entropy generation problem. More exactly, in this work, the focus is on entropy generation due to highly accelerated plate motion in the vertical direction which is not studied in the literature before this. Water is taken as a counter-example of a Newtonian fluid. The Prandtl number for water is taken as 6.2, however, just for variation purpose, some other values are also taken. The problem is first modeled and then the dimensionless analysis is used to get a transformed system. The results problem is solved for the exact solution using the Laplace transform method whereas for numerical technique, the numerical scheme known as finite difference method has been used. Results are computed and then plotted in various plots and discussed in detail. This paper ends with a conclusion at the end.

### Description of the problem

Consider the Casson fluid model for the flow of sodium alginate solution over as accelerated plated. Meanwhile, it is assumed at $\tau \le 0$, the system was at ambient temperature with zero velocity. However, at $\tau = 0^{ + }$, the fluid starts motion with $v\left( {0,\tau } \right) = A\tau^{2}$, and temperature variated to $\vartheta \left( {0,\tau } \right) = \theta_{w}$. Hence, convection is taken place. The governing equation of the flow phenomena are given by

1 $$ \rho \frac{\partial v(\eta ,\tau )}{{\partial \tau }} = \mu \frac{{\partial^{2} v(\eta ,\tau )}}{{\partial \eta^{2} }} + \rho g\beta_{0} (\theta (\eta ,\tau ) - \theta_{\infty } ) - \delta B_{0}^{2} v(\eta ,\tau ) $$

the energy equation in the absence of radiation is presented as under.

2 $$ \rho c_{p} \frac{\partial \theta (\eta ,\tau )}{{\partial \tau }} = k\frac{{\partial^{2} \theta (\eta ,\tau )}}{{\partial \eta^{2} }}, $$

subject to

3 $$ \left. {\begin{array}{*{20}l} {v\left( {\eta ,0} \right) = 0,} \hfill & {\quad \theta \left( {\eta ,0} \right) = \theta_{\infty } } \hfill \\ {v\left( {0,\tau } \right) = A\tau^{2} ,} \hfill & {\quad v\left( {\infty ,\tau } \right) = 0} \hfill \\ {\theta \left( {0,\tau } \right) = \theta_{w} ,} \hfill & {\quad \theta \left( {\infty ,\tau } \right) = \theta_{\infty } } \hfill \\ \end{array} } \right\}, $$

The below given dimensionless variables

$$ v^{*} = \frac{v}{{\left( {v^{2} A} \right)^{\frac{1}{5}} }},\, \, \eta^{*} = \frac{{\eta A^{\frac{1}{5}} }}{{\nu^{\frac{3}{5}} }},\, \, \tau^{*} = \frac{{\tau A^{\frac{2}{5}} }}{{\nu^{\frac{1}{5}} }},\,\,\,\,\theta^{*} (\eta ,\tau ) = \frac{{\theta - \theta_{\infty } }}{{\theta_{w} - \theta_{\infty } }}, $$

are incorporated into Eqs. (1–3). The momentum equation is given in Eq. (1) is nondimensionalized and is presented as under.

4 $$ \frac{\partial v(\eta ,\tau )}{{\partial \tau }} = \frac{{\partial^{2} v(\eta ,\tau )}}{{\partial \eta^{2} }} + Gr\theta (\eta ,\tau ) - H_{a} v(\eta ,\tau ), $$

The heat equation, which was displayed in dimensional form as Eq. (2) is now given in nondimensional form as under.

5 $$ \Pr \frac{\partial \theta }{{\partial \tau }} = \frac{{\partial^{2} \theta }}{{\partial \eta^{2} }}. $$

With non-similar initial and boundary conditions as:

6 $$ \left. {\begin{array}{*{20}l} {v\left( {\eta ,0} \right) = 0,} \hfill & {\quad v\left( {0,\tau } \right) = \tau^{2} ,} \hfill & {\quad v\left( {\infty ,\tau } \right) = 0} \hfill \\ {\theta \left( {0,\tau } \right) = 1,} \hfill & {\quad \theta \left( {\infty ,\tau } \right) = 0} \hfill & {\quad \theta \left( {\eta ,0} \right) = 0} \hfill \\ \end{array} } \right\}, $$

where

$$ Gr = \frac{{g\beta_{\theta } \Delta \theta }}{{A^{\frac{3}{5}} \upsilon^{\frac{1}{5}} }}, \, H_{a} = \frac{\delta }{\rho }B_{0}^{2} A^{{ - \frac{2}{5}}} \upsilon^{\frac{1}{5}} , \, \Pr = \frac{{\mu c_{p} }}{k} $$

### Entropy generation

In heat transfer systems, using Eq. (4–6), the reduction in energy losses can be mathematically expressed^4–6,13,14^.

7 $$ E_{gen} = \frac{K}{{\theta_{\infty }^{2} }}\left( {\frac{\partial \theta }{{\partial \eta }}} \right)^{2} + \frac{\mu }{{\theta_{\infty } }}\left( {\frac{\partial v}{{\partial \eta }}} \right)^{2} + \frac{{\delta \beta_{0}^{2} }}{{\theta_{\infty } }}v^{2}. $$

Taking into consideration, the non-similarity variable, $\partial \theta /\partial \eta = \Delta \theta A^{\frac{1}{5}} \nu^{{ - \,\frac{3}{5}}} \partial \theta^{*} /\partial \eta^{*}$ and $\partial v/\partial y = A^{\frac{2}{5}} \nu^{{ - \,\frac{1}{5}}} \partial v^{*} /\partial \eta^{*}$ are produced and incorporated into Eq. (7), which yields

8 $$ N_{s} = \frac{{E_{gen} }}{{E_{0} }} = \left[ {\left( {\frac{\partial \theta }{{\partial \eta }}} \right)^{2} + \frac{{B_{r} }}{\Omega }\left( {\frac{\partial v}{{\partial \eta }}} \right)^{2} + \frac{{B_{r} }}{\Omega }H_{a} v^{2} } \right], $$

where

$$ N_{s} = \frac{{E_{gen} \nu^{\frac{6}{5}} \theta^{2}_{\infty } }}{{kA^{2/5} (\Delta \theta )^{2} }},\,Br = \frac{{\mu A^{\frac{2}{5}} \nu^{\frac{4}{5}} }}{k\Delta \theta },\,\,\Omega = \frac{\Delta \theta }{{\theta_{\infty } }} = \frac{{\theta_{w} - \theta_{\infty } }}{{\theta_{\infty } }}. $$

### Bejan number

The Bijan number for the dimensionless system developed in Eqs. (4–6) is defined as

9 $$ B_{e} = \frac{{\frac{K}{{\theta_{\infty }^{2} }}\left( {\frac{\partial \theta }{{\partial \eta }}} \right)^{2} }}{{\frac{K}{{\theta_{\infty }^{2} }}\left( {\frac{\partial \theta }{{\partial \eta }}} \right)^{2} + \frac{\mu }{{\theta_{\infty } }}\left( {\frac{\partial v}{{\partial \eta }}} \right)^{2} + \frac{{\delta \beta_{0}^{2} }}{{\theta_{\infty } }}v^{2} }} $$

and

10 $$ B_{e} = \frac{{\left( {\frac{\partial \theta }{{\partial \eta }}} \right)^{2} }}{{\left( {\frac{\partial \theta }{{\partial \eta }}} \right)^{2} + \frac{{B_{r} }}{\Omega }\left( {\frac{\partial v}{{\partial \eta }}} \right)^{2} + \frac{{B_{r} }}{\Omega }H_{a} v^{2} }} $$

### Exact solutions by Laplace transform method

In the literature, numerical or approximate methods are used to deal with mixed convection problems, and the exact solutions are rare. Here, the exact solution can be obtained via the Laplace transformation method, (4–6) gives by applying the Laplace transformation:

11 $$ q\overline{v}(\eta ,q) = \frac{{\partial^{2} \overline{v}(\eta ,q)}}{{\partial \eta^{2} }} + Gr\overline{\theta }(\eta ,q) - H_{a} \overline{v}(\eta ,q) $$

The initial condition is used while applying the integral transform and the boundary conditions in the transformed variable are given as under.

12 $$ \overline{v}\left( {0,q} \right) = \frac{2}{{q^{3} }},\,\,\,\,\,\,\overline{v}\left( {\infty ,q} \right) = 0 $$

In the transformed variable the energy equation whit boundary conditions are given as:

13 $$ \Pr q\overline{\theta }\left( {\eta ,q} \right) = \frac{{\partial^{2} \overline{\theta }(\eta ,q)}}{{\partial \eta^{2} }} $$

14 $$ \overline{\theta }\left( {0,q} \right) = \frac{1}{q},\,\,\,\,\,\,\overline{\theta }\left( {\infty ,q} \right) = 0 $$

Equation (13) can be solved by using boundary conditions (14) as:

15 $$ \overline{\theta }\left( {\eta ,q} \right) = \frac{{e^{{ - \eta \sqrt {\Pr q} }} }}{q} $$

The inverting the Laplace transform is employed which yield to

16 $$ \theta \left( {\eta ,\tau } \right) = erfc\left( {\frac{{\eta \sqrt {\Pr } }}{2\sqrt \tau }} \right). $$

Meanwhile, Eq. (11) is solved taking into account Eqs. (11, 12, 15) yield to

17 $$ \overline{v}(\eta ,q) = \frac{2}{{q^{3} }}e^{{ - \eta \sqrt {q + \text{H}_{a}} }} - \frac{a}{{q\left( {q + a_{0} } \right)}}e^{{ - \eta \sqrt {q + \text{H}_{a}} }} + \frac{a}{{q\left( {q + a_{0} } \right)}}e^{{ - \eta \sqrt {\Pr } \sqrt q }} $$

where $$\text{Pr}\neq {1}$$.

$$ a = \frac{Gr}{{1 - \Pr }}, \, a_{0} = \frac{\text{H}_{a} }{{1 - \Pr }}. $$

with the inverse Laplace transform,

18 $$ v(\eta ,\tau ) = v_{1} (\eta ,\tau ) + v_{2} (\eta ,\tau ) + v_{3} (\eta ,\tau ) $$

where

19 $$ \begin{aligned} v_{1} (\eta ,\tau ) & = 2\left[ {\left( {\frac{{\tau^{2} }}{4} - \frac{\eta }{16}\sqrt {\frac{1 }{\text{H}}_{a}} } \right)e^{{ - \eta \sqrt {\text{H}_{a}} }} erfc\left( {\frac{\eta }{2}\sqrt {\frac{1 }{\tau }} - \sqrt {\text{H}_{\text{a}}\tau } } \right) - \left( {\frac{{\tau^{2} }}{4} + \frac{\eta }{16}\sqrt {\frac{1 }{\text{H}}_{a}} } \right)e^{{\eta \sqrt {\text{H}_{a}} }} erfc\left( {\frac{\eta }{2}\sqrt {\frac{1 }{\tau }} + \sqrt {\text{H}_{a}\tau } } \right)} \right]  \\ \end{aligned} $$$$ v_{2} (\eta ,\tau ) = \frac{a}{{a_{0} }}\left[ \begin{gathered} \frac{{e^{{ - a_{0} \tau }} }}{2}\left( {e^{{ - \eta \sqrt {\left( {\text{H}_{a} - a_{0} } \right)} }} erfc \, \left( {\frac{\eta }{2}\sqrt {\frac{1 }{\tau }} - \sqrt {\left( {\text{H}_{a} - a_{0} } \right)\tau } } \right) + e^{{\eta \sqrt {\left( {\text{H}_{a} - a_{0} } \right)} }} erfc \, \left( {\frac{\eta }{2}\sqrt {\frac{1 }{\tau }} + \sqrt {\left( {\text{H}_{a} - a_{0} } \right)\tau } } \right)} \right) \quad \hfill \\ -\frac{1}{2}\left( {e^{{ - \eta \sqrt {\text{H}_{a}} }} erfc \, \left( {\frac{\eta }{2}\sqrt {\frac{1 }{\tau }} - \sqrt {\text{H}_{a}\tau } } \right) + e^{{\eta \sqrt {\text{H}_{a}} }} erfc \, \left( {\frac{\eta }{2}\sqrt {\frac{1 }{\tau }} + \sqrt {\text{H}_{a}\tau } } \right)} \right) \hfill \\ \end{gathered} \right] $$$$ v_{3} (\eta ,\tau ) = \frac{a}{{a_{0} }}\left[ {erfc \, \left( {\frac{\eta }{2}\sqrt {\frac{\Pr }{\tau }} } \right) - \frac{{e^{{a_{0} \tau }} }}{2}\left( {e^{{ - \eta \sqrt { - a_{0} \Pr } }} erfc \, \left( {\frac{\eta }{2}\sqrt {\frac{\Pr }{\tau }} - \sqrt { - a_{0} \tau } } \right) + e^{{\eta \sqrt { - a_{0} \Pr } }} erfc \, \left( {\frac{\eta }{2}\sqrt {\frac{\Pr }{\tau }} + \sqrt { - a_{0} \tau } } \right)} \right)} \right] $$

### Skin friction

The skin friction of the system in dimensionless form is given by

20 $$ c_{f} = \left. {\frac{\partial v(\eta ,\tau )}{{\partial \eta }}} \right|_{\eta = 0} $$

### Nusselt number

From Eq. (15) the Nusselt number in e dimensionless form is given by

21 $$ Nu = \left. {\frac{\partial \theta (\eta ,\tau )}{{\partial \eta }}} \right|_{\eta = 0} $$

## Results and discussion

A new problem of entropy generation is studied in this work numerically as well as analytically. The flow is considered over quadratic accelerated plate in the presence of a strong magnetic field. Analytical part of this work is done via Laplace transform whereas the numerical part is done using the finite difference scheme. Results for different parameters are computed and displayed in various plots and tables. The finite difference scheme results are computed using MATLAB.

### Finite difference scheme

Finite difference method is a distinguished technique in the numerical analysis. This technique is applied here to compute results shown in tabular form. In this condition, the numerical solution which is identified exclusively at a finite number of points in the physical field is recognized as discrete. The operator of this numerical technique can pick the number of those points. In fact, both resolution and numerical solution precision are enhanced when the point number rises. The discrete approximation leads to a group of algebraic equations which are assessed according to the values of the discrete unknowns.

The assortment of positions where the discrete solution is calculated is recognized as the mesh. The fundamental basis of the finite-difference method is to substitute continuous derivatives by the supposed difference formulas which include only the discrete values related to locations on the mesh. The application of the finite-difference method to a differential equation implicates substituting all derivatives with difference formulas. Both derivatives with respect to space and derivatives with respect to time exist in the heat equation. Several systems can be obtained after varying the arrangement of mesh points in the difference formulas. Therefore, The numerical solution achieved via whatever valuable scheme will approximate the real solution to the original differential equation.

The above finite difference scheme, has been used for computing the following tabulated data. Therefore, Tables 1, 2, 3 and 4 are formed. The numerical values given in Table 1 shows the variation in velocity of the fluid. These results shows that increasing values of Ha causes the velocity water to decrease. However, increasing Gr decreases fluid velocity and the fluid flows slowly. Variation in the skin-friction for different values of Ha and Gr are shown in Table 2. From this table we can clearly see that increasing Ha results a decrease in the magnitude of skin-friction. However, skin-friction increases for larger values of Gr. Table 3 shows variation in the Bejan number for Ha and Gr when other parameters are kept constant. With increasing Ha, Bejan number increases but decreases for increasing Gr. The finite difference results for entropy generation are shown in Table 4. This table clearly shows that with increasing Ha, entropy generation decreases. However, for larger values of Br and $\Omega$, entropy generation increases.

**Table 1:** Finite difference results for velocity.

Ha	Gr	V
0.5	1	0.4067
–	–	0.3562
1	–	0.3919
0.5	–	0.4937
–	2	0.0190

**Table 2:** Finite difference results for skin-friction.

Ha	Gr	$c_{f}$
0.5	1	0.0105
–	–	0.0094
1	–	0.0106
0.5	–	0.0105
–	2	0.0141

**Table 3:** Finite difference results for Bejan number.

Ha	Gr	Be
0.5	1	$1.0961 \times e^{ - 6}$
–	–	$1.9870 \times e^{ - 6}$
1	–	$1.1753 \times e^{ - 6}$
0.5	–	$9.8233 \times e^{ - 7}$
–	2	$1.0484 \times e^{ - 6}$

**Table 4:** Finite difference results for entropy generation.

Ha	$\tau$	Br	$\Omega$	Pr	Gr	η	Ns
0.5	2	0.5	10	6.2	1	0.5	0.1907
1	–	–	–	–	–	–	0.1878
0.5	–	0.9	–	–	–	–	0.343
–	–	0.5	15	–	–	–	0.1272
–	–	–	10	–	–	–	0.1985

### Numerical simulations of exact solutions

**Figure 1:**
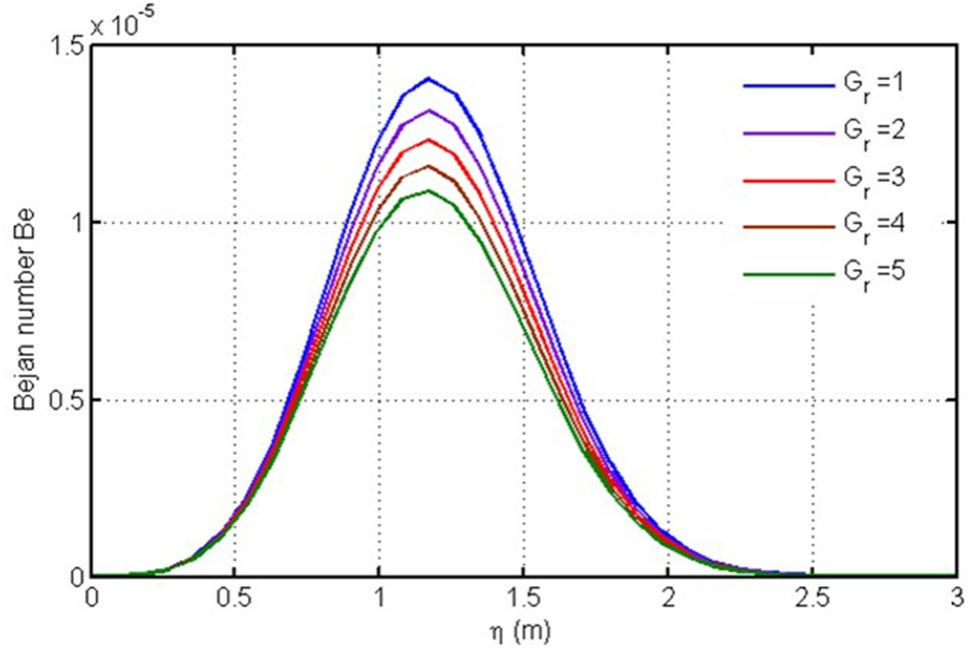
Plot for Bejan number versus $\eta$ against $Gr$.

In Fig. 1, Bejan number is plotted against the space variable $\eta$ for different values of Grashof number. It is clear from this figure that Gr is responsible for lowering Bejan number.

**Figure 2:**
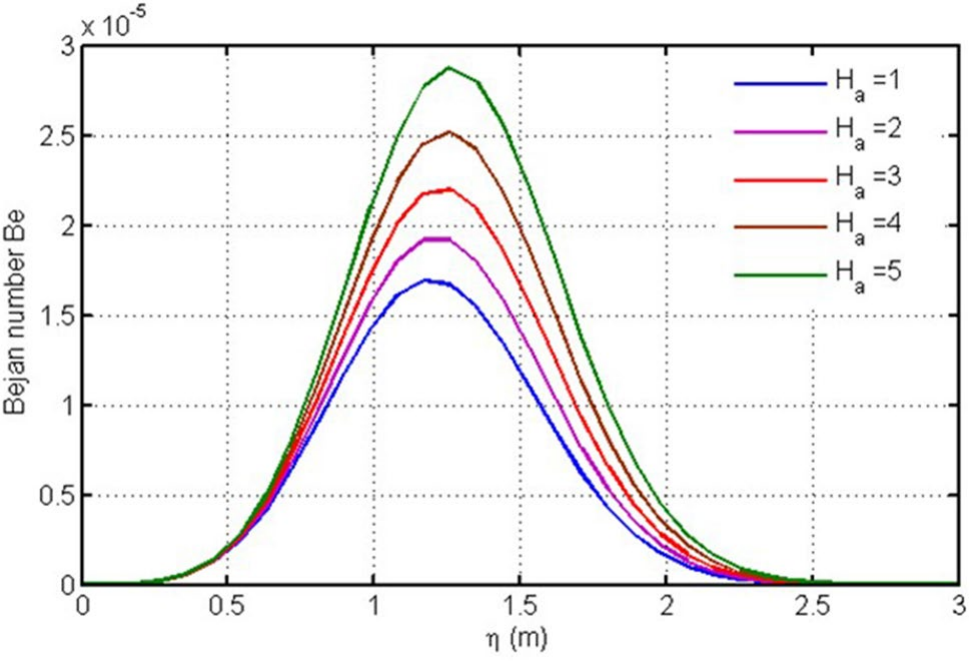
Plot for Bejan number versus $\eta$ against $Ha.$

In the subsequent figure (Fig. 2), Bejan number versus $\eta$ is examined for magnetic parameter (Ha), also knows as Hartmann number. It is found that Ha cause Bejan number to grow, however, the increase in Bejan number due to Ha is more dominant.

**Figure 3:**
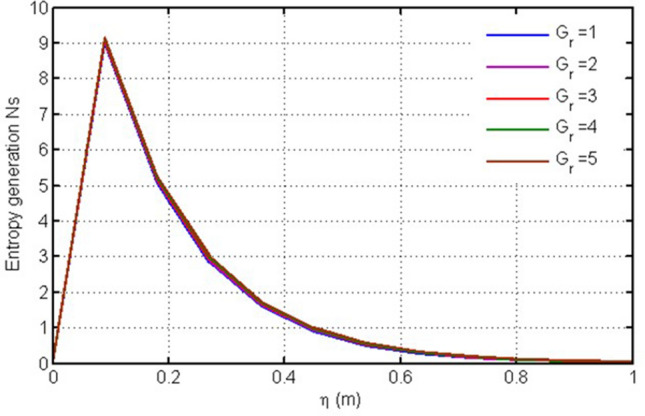
Plot for entropy generation versus $\eta$ against $Gr.$

**Figure 4:**
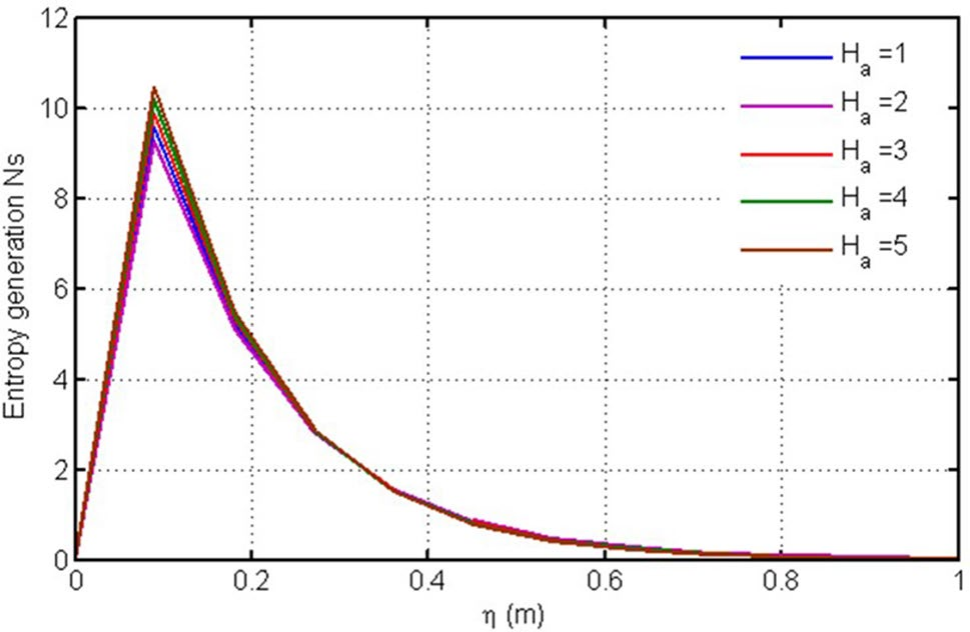
Plot for entropy generation versus $\eta$ against $Ha.$

Figure 3 shows plot for entropy generation versus $\eta$ against $Gr.$ In this figure the variation in entropy generation profile is shown for different values of Thermal Grashof number, the greater this value, the higher is the entropy generation and vice versa. Physically the higher values $Gr$ enhances the irreversibility. Figure 4 is plotted for entropy generation versus $\eta$ against Ha. Entropy generation behaves in opposite manner for the larger values of Ha i.e. decreasing with increasing Ha (Fig. 4) due to prominent friction force.

**Figure 5:**
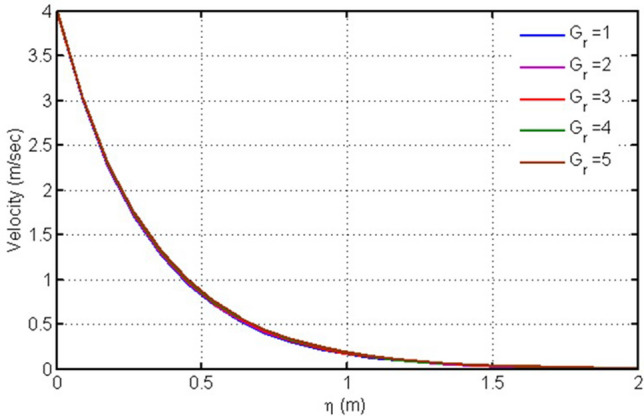
Plot for velocity versus $\eta$ against $Gr.$

The velocity profile for different values of Gr is plotted in this figure (Fig. 5). Due to buoyancy forces the velocity decreases with the increasing values of Grashof number. This purpose is served by varying the Grashof number (Gr) while other parameters possess some constant values. Gr is a critical quantity in those ow problems which involve the free convection mechanism. The physical phenomenon causing such results is an augmentation in the wall temperature due to a rise in Gr. This leads to reduce the force of internal resistance and makes gravitational effects more strong. Correspondingly, the viscous influence on the velocity is efficiently encountered by the buoyancy force and the flow field species a rising trend.

**Figure 6:**
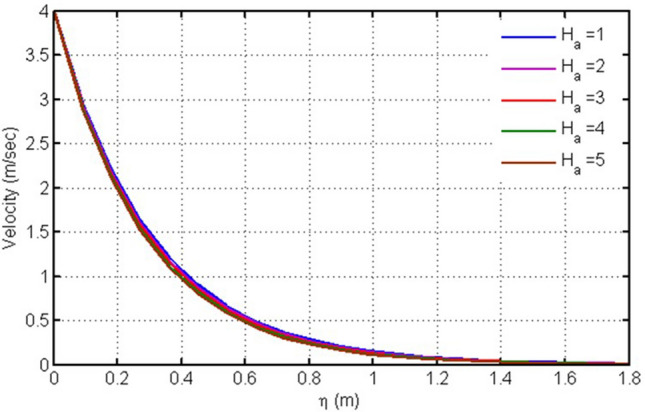
Plot for velocity versus $\eta$ against $Ha.$

Figure 6 is plotted to illustrate the velocity field for several values of the magnetic parameter Ha, (Hartmann number). It is noticed that Ha produces decelerating effects on the fluid motion. Physically, Newtonian fluid acquires maximum transportation speed in the absence of magnetic influence. The physical argument that justifies this flow retardation is the origination of the Lorentz force. According to Lorentz's theory, this force is a resistive one, which serves against the flow direction. Moreover, as Ha increases, this force enhances the viscous effects and drags the viscous fluid in a reverse direction.

**Figure 7:**
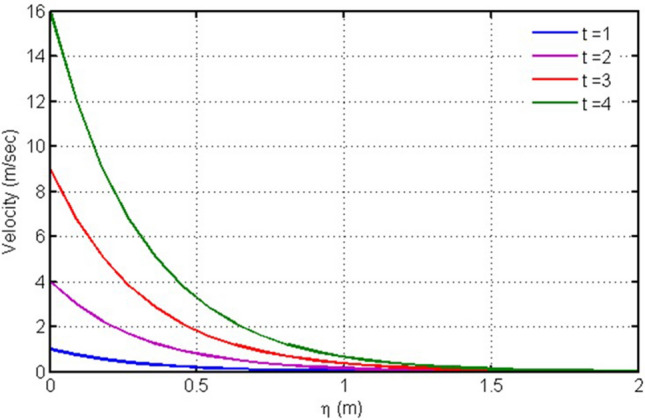
Plot for velocity versus $\eta$ against $time.$

Figure 7 is plotted for velocity versus $\eta$ against $time.$ It is found that velocity increases with increasing time near the plate, however, for values away from the plate, goes to zero.

**Figure 8:**
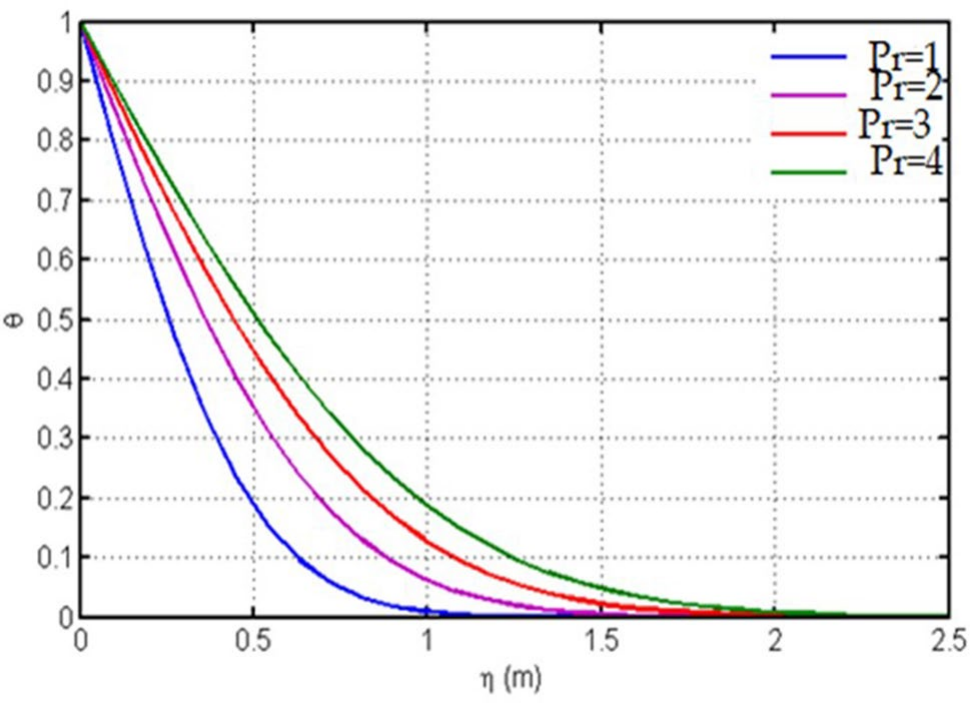
Plot for temperature versus $\eta$ against $\mathrm{Pr}.$

Figure 8 is plotted for temperature versus $\eta$ against $\mathrm{Pr}.$ For different values of time this figure is plotted. Here it is noticed that the obtained solutions are satisfying the imposed conditions on the boundary. For different Pr the temperature profile is drawn in this figure. From Pr = 1 to Pr = 4 the temperature in showing increasing behavior.

**Figure 9:**
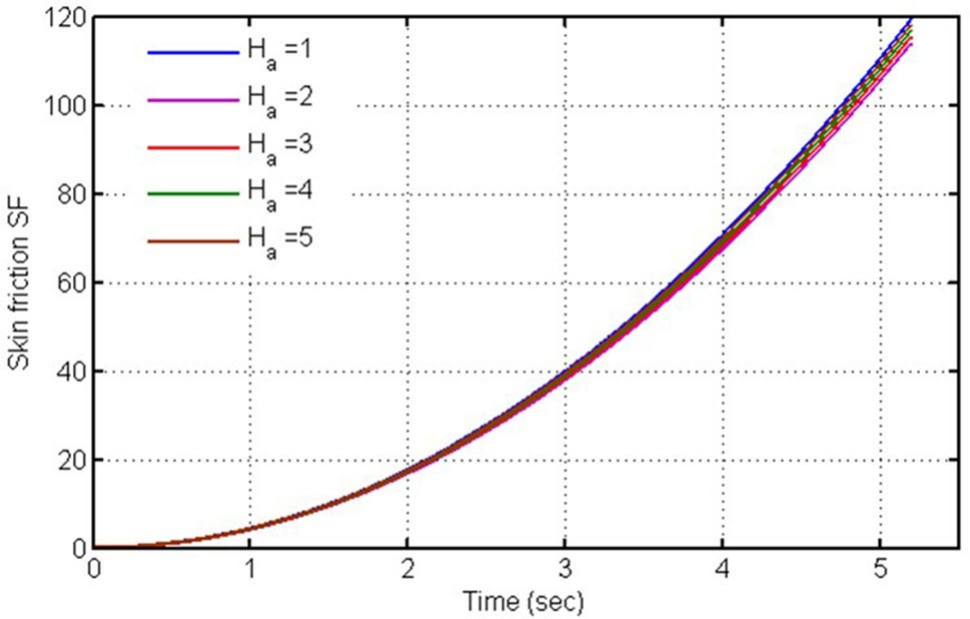
Plot for skin-friction versus time against $\mathrm{H}a.$

**Figure 10:**
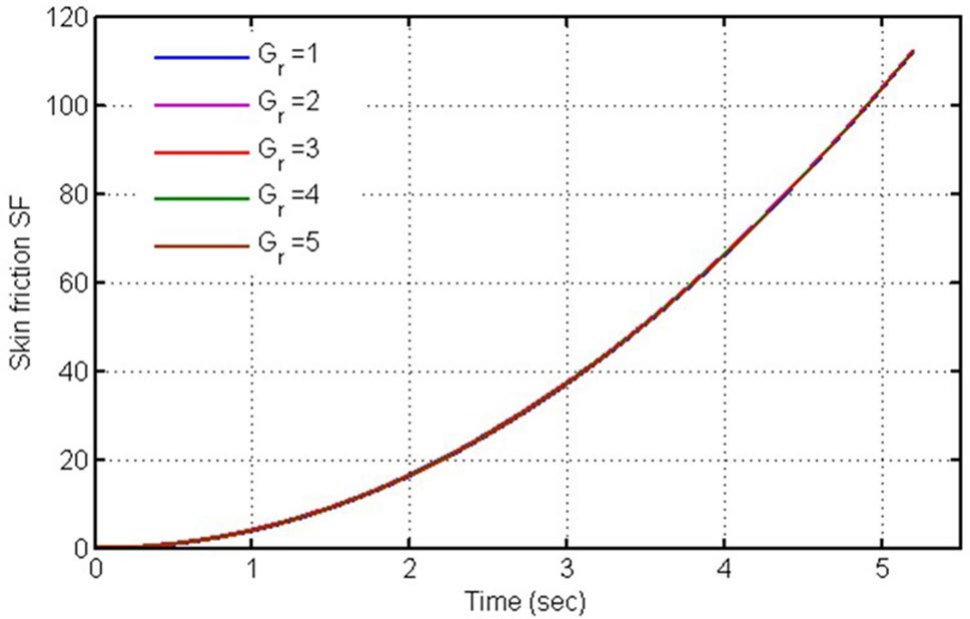
Plot for skin-friction versus time against $Gr.$

The skin friction against time (t) is plotted in this figure (Fig. 9) for various values of $Ha$, it is clearly seen from this plot that skin friction is decreasing for increasing values of $Ha.$ Fig. 10 is plotted for skin-friction versus time against $Gr,$ when other parameters possess some constant values. Gr is a critical quantity in such a flow problem which involves the free convection mechanism, and with its increasing values skin-friction decreases. Physically, for different values of Gr the skin friction is showing decreasing behavior as contrary to velocity due to weaker contact. However, the change is very small to clearly differentiate.

**Figure 11:**
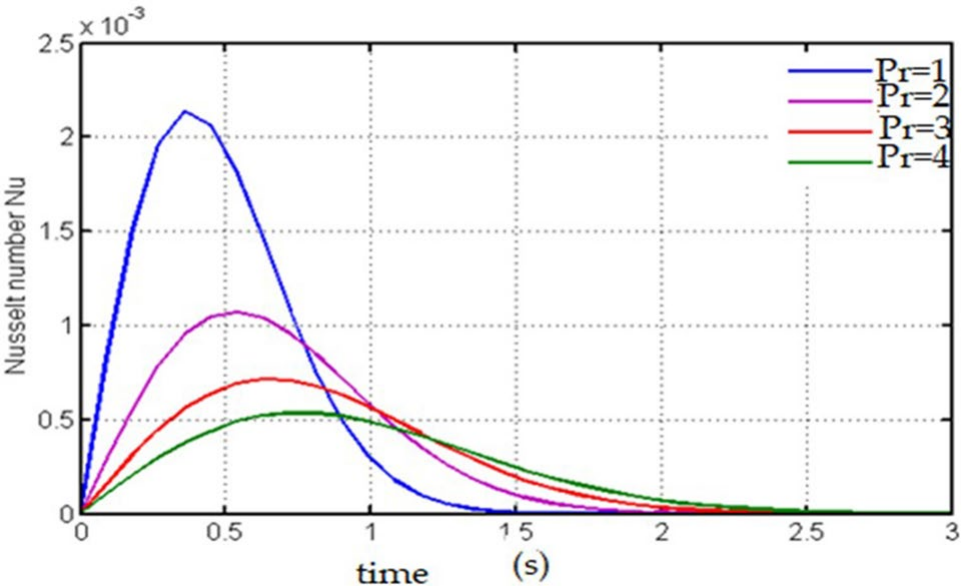
Plot for Nusselt number versus time against $\mathrm{Pr}.$

For various values of Pr the Nusselt number is drawn in this figure (Fig. 11) against time. Near the plate the Nusselt number decreases with increasing values of time while away from the plate the Nusselt number increases with increasing values of Pr.

## Concluding remarks

In this work, the effect of magnetic field is studied on highly accelerated fluid motion and the the entropy generation analysis is then performed by taking water as a Newtonian fluid. The fluid is constantly heated from one side and heat transfers due to convection. The problem is first modelled and then solved for exact and numerical solutions. Results for Bejan number, entropy generation, velocity, temperature, and skin-friction are computed in tables and various plots. The following key points are concluded from this work.

- Entropy generation decreases with increasing Ha but increases for larger values of Br and $\Omega$.
- Ha reduces fluid motion.
- With increasing Ha, Bejan number increases but decreases for increasing Gr.
- The variation in the Bejan number is more visible compare to entropy generation for larger values of Gr.
- The magnitude of entropy generation is bigger compare to Bejan number for greater Hartman number, however, this change in Bejan number is more effective.

## List of symbols

*A*: Acceleration of the plate, (ms^−2^)
*v*: Velocity of the fluid, (ms^−1^)
$\vartheta$: Temperature of the fluid, (K)
$g$: Acceleration due to gravity, (ms^−2^)
$B_{0}$: Applied magnetic field
$c_{p}$: Specific heat at a constant pressure, (J kg^−1^ K^−1^)
$Gr$: Thermal Grasshof number, (–)
$k$: Thermal conductivity of the fluid, (W m^−2^ K^−1^)
$$Nu$$: Nusselt number, (–)
$$\Pr$$: Prandtle number, $$( = \mu c_{p} /k)$$
$q$: Laplace transforms parameter

## Greek symbols

$\mu$: Dynamic viscosity, (kg m^−1^-s^−1^)
$\rho$: Fluid density, (kg ms^−3^)
$\beta_{0}$: The volumetric coefficient of thermal expansion, (K^−1^)
$$\tau$$: Time, (s)
$\theta$: Fluid temperature far away from the plate, (K)
$\sigma$: Stephen-Boltzmann constant
